# Effects of fulvic acid on growth performance, serum index, gut microbiota, and metabolites of Xianju yellow chicken

**DOI:** 10.3389/fnut.2022.963271

**Published:** 2022-08-05

**Authors:** Peishi Feng, Qiaoqiao Li, Hanxue Sun, Jinfeng Gao, Xuan Ye, Yi Tao, Yong Tian, Ping Wang

**Affiliations:** ^1^College of Pharmaceutical Sciences, Zhejiang University of Technology, Hangzhou, China; ^2^Institute of Animal Husbandry and Veterinary Medicine, Zhejiang Academy of Agricultural Sciences, Hangzhou, China; ^3^Xianju Breeding Chicken Farm, Taizhou, China

**Keywords:** gut microbiota, growth performance, inflammation, Xianju yellow chicken, fulvic acid

## Abstract

Fulvic acid (FA) is a mixture of polyphenolic acid compounds extracted from humus, peat, lignite, and aquatic environments; it is used in traditional medicine to treat digestive tract diseases. The purpose of the present study was to investigate the effect of FA on growth performance, inflammation, intestinal microbiota, and metabolites in Xianju yellow chicken. The 240 Xianju yellow chickens (age, 524 days) included were randomly categorized into 4 treatments with 6 replicates per treatment and 10 birds per replicate. Birds received a basal diet or a diet supplemented with 500, 1,000, or 1,500 mg/kg of FA, for a period of 42 days. Dietary supplementation of FA improved average daily gain (ADG) and feed conversion ratio (FCR) (*P* > 0.05). Compared with the control group, the serum level of TNF-α in birds supplemented with FA was significantly decreased (*P* < 0.05), and that of IL-2 was significantly increased after administration of 1,500 mg/kg FA (*P* < 0.05). Analysis of gut microbiota indicated that FA reduced the relative abundance of genus *Mucispirillum, Anaerofustis*, and *Campylobacter*, but enriched genus *Lachnoclostridium, Subdoligranulum, Sphaerochaeta, Oscillibacter*, and *Catenibacillus* among others. Untargeted metabolomic analyses revealed that FA increased 7-sulfocholic acid, but reduced the levels of Taurochenodeoxycholate-7-sulfate, LysoPC 20:4 (8Z, 11Z, 14Z, 17Z), LysoPC 18:2, Phosphocholine and other 13 metabolites in the cecum. The results demonstrated that FA may potentially have a significant positive effect on the growth performance and immune function of Xianju yellow chicken through the modulation of the gut microbiota.

## Introduction

Using antibiotics in livestock and poultry farming could efficiently reduce disease frequency, improve growth performance, and enhance feeding efficacy. However, overuse of antibiotics in livestock and poultry promotes the development of antibiotic resistance, which has garnered considerable attention. Currently, several countries have banned the use of antibiotics as growth promoters in poultry farming. The general tendency of the reduction in the use of antibiotics in the breeding industry has created a need for alternative strategies. Chinese herbs, which have beneficial nutritional and medicinal properties, can potentially be used as feed additives to improve the performance of animal growth owing to their strong antimicrobial activity and absence of residues.

Fulvic acid (FA) is a mixture of polyphenolic acid compounds extracted from humus, peat, lignite, and aquatic environments; it is characterized by its small molecular weight and the abundance of biologically active molecules (1, 2). FA, confirmed as the main active component of Wujinshi, was considered an effective treatment for ulcerative carbuncle in the Compendium of Materia Medica. Modern pharmacological studies have revealed that FA has multiple effects, including antioxidant (3), anti-inflammatory (4), immunomodulatory (5), and antidiabetic (6). In recent years, studies have demonstrated that adding FA to the feed or drinking water could enhance the growth rate, improve feed conversion ratio, and increase immunity (7, 8). However, using FA as an alternative antimicrobial feed additive in animal production remains in its infancy.

Xianju yellow chicken is one of the local breeds in the Zhejiang province, China. This breed of chicken is characterized by its superior meat quality, high egg production, and strong adaptability. In the poultry industry, high stocking density and intensive feeding have resulted in a decline in immunity, decrease in productivity, and low feed conversion ratio. FA has been used as a traditional Chinese medicine owing to its immune regulatory activity. However, the effects of dietary supplementation with FA require thorough examination in terms of the poultry industry. Therefore, the purpose of this study was to investigate the suitable supplemental amount of FA in poultry feed. Furthermore, the influence of FA on the gut microbiota and potential mechanisms underlying the regulation of productive performance through FA supplementation in poultry farming has been explored.

## Materials and Methods

The experimental procedures for this study were approved by the Ethics Committee of the Zhejiang Academy of Agricultural Sciences and the Ministry of Science and Technology of the People's Republic of China.

### Animals, housing, and experimental treatments

A total of 240 (age, 524 days; sex, female) Xianju yellow chickens of similar egg production rates and weight (1.83 ± 0.33 g) were provided by Xianju Breeding Chicken Farm, Zhejiang province, China. All chickens were randomly divided into 4 groups with 6 replicates each, and each replicate included 10 birds. The chickens in the Control group were fed a basal diet, and the other 3 groups were, respectively, fed the basal diet supplemented with 500 mg/kg (FA-L), 1,000 mg/kg (FA-M), and 1,500 mg/kg FA (FA-H) for 42 days. The FA provided by Slona Biological Technology Co., Ltd (the content of FA was 19.28%, Supplementary Table 1). The basal diet had been formulated according to the nutritional requirements of yellow chickens, which has been presented in Table 1. All chickens were raised in three-story cages (two chickens per cage) under the recommended environmental conditions and were provided access to feed and water *ad libitum*. Feed consumption per cage was recorded every 10 days. The chickens were weighed at the end of the experiment. Egg number were recorded daily. Average daily gain (ADG), average daily feed intake (ADFI), feed conversion ratio (FCR), laying rates, and feed/egg were determined for the overall rearing period.

**Table 1:** Formulation and composition of the basal diet.

**Item**	**Value**
**Ingredients (%)**	
Corn	60.80
Soybean meal	12.60
Extruded soybean	10.00
Corn gluten meal	3.00
Limestone	10.50
Calcium hydrogen phosphate	1.00
Soybean oil	0.40
Lysine sulfate	0.40
DL- methionine	0.30
Salt	0.39
Choline chloride	0.17
Vitamin and trace mineral premix^a^	0.44
**Nutrition levels** ^**b**^	
Metabolizable energy (MJ/kg)	11.43
Crude protein (%)	15.99
Moisture (%)	11.84
Ca (%)	4.30
Total phosphorus (%)	0.48
Non-phytate phosphorous (%)	0.30
Lysine (%)	0.93
Methionine and cystine (%)	0.71

a * Supplied per kg feed: vitamin A, 12,000 IU; vitamin D_3_, 2,000 IU; vitamin E, 30 IU; vitamin K_3_, 2 mg; vitamin B_1_, 2 mg; vitamin B_2_, 8 mg; vitamin B_6_, 5 mg; vitamin B_12_, 0.010 mg; biotin, 0.25 mg; calcium pantothenate, 12 mg; nicotinic acid, 50 mg; folic acid, 2 mg; Fe, 80 mg; Mn, 55 mg; Zn, 75 mg; Cu, 7 mg; I, 0.5 mg; Se, 0.15 mg. ^b^ Nutrition levels were calculated values*.

### Sampling and measurements

At the end of the experiment (the 42nd day), two chickens were randomly selected from each replicate, individually weighed, and exsanguinated. Blood samples were collected from the jugular vein of each bird without the use of anticoagulants. One chicken was used to measure and calculate the slaughter performance. Furthermore, the lengths of the duodenum, jejunum, ileum, and ceca were measured. Digesta from the ceca of another chicken were mixed and divided into 2 subsamples; these subsamples were then placed in liquid nitrogen and stored at −80°C for analyses of gut microbiota and untargeted metabolomics.

### Slaughter performance and meat quality traits determination

According to the poultry production performance noun terms and metric statistics method (NY/T823-2020), the live weight before slaughter, dressed weight, half-eviscerated weight with giblet, eviscerated weight, leg muscle weight, breast muscle weight, and abdominal fat weight were measured and the dressed percentage, percentage of half-eviscerated weight with giblet, percentage of eviscerated yield, percentage of leg muscle yield, percentage of breast muscle yield and percentage of abdominal fat yield were calculated. The formulae for calculating the abovementioned parameters have been described below.


$$\begin{array}{c} Dressed\;percentage\;=\frac{Dressed\;weight}{Live\;weight\;before\;slaughter}\times 100\\ Percentage\;of\;half\text{-}eviscerated\;yield\;with\;giblet\;=\frac{Half\text{-}eviscerated\;weight\;wiht\;giblet}{Live\;weight\;before\;slaughter}\times 100\\ Percentage\;of\;eviscerated\;yield\;=\frac{Eviscerated\;weight}{Live\;weight\;before\;slaughter}\times 100\\ Percentage\;of\;leg\;muscle\;yield\;=\frac{Leg\;muscle\;weight}{Eviscerated\;weight}\times 100\\ Percentage\;of\;breast\;muscle\;yield\;=\frac{Breast\;muscle\;weight}{Eviscerated\;weight}\times 100\\ Percentage\;of\;abdominal\;fat\;yield\;=\frac{Abdomianl\;fat\;weight}{Eviscerated\;weight+Abdomianl\;fat\;weight}\times 100 \end{array}$$

After weighing, breast muscles were measured *pH* at 45 min in triplicate with a PH-STAR pH meter (Matthaus, German). And meat color was measured using a colorimeter (CE-410, Konica Minolta Sensing, Inc., Osaka, Japan).

### Serum cytokines measurement

The collected blood samples, which were allowed to stand for 30 min to coagulate, were centrifuged at 3,500 rpm for 10 min at 4°C to obtain the serum. Serum levels of the cytokines such as IL-2, IL-6, IL-10, IFN-α, and TNF-α were measured using the ELISA kit (Shanghai Hepeng Biological Co., Ltd, China) according to the manufacturer's protocol.

### Serum liver function indexes

The serum liver function indexes, including alanine aminotransferase (ALT), aspartate aminotransferase (AST), and total protein (TP) were measured using an automatic biochemical analyzer (Hitachi 7020, Tokyo, Japan).

### Gut microbiota analysis

DNA from ceca digesta samples of the Control and FA-H group were extracted using the E.Z.N.A. ^®^Stool DNA Kit (D4015, Omega, Inc., USA) according to the manufacturer's instructions. The total DNA was eluted in 50 μL of elution buffer and stored at −80°C until measurement in the PCR by Majobio Bio-pharm Technology Co., Ltd (Shanghai, China). The V3–V4 regions of 16S rDNA were amplified using 341F (5′-CCTACGGGNGGCWGCAG-3′) and 806R (5′- GGACTACHVGGGTWTCTAAT-3′) (9). PCR amplification was performed in a total volume of 25 μL reaction mixture containing 25 ng of template DNA, 12.5 μL PCR Premix, 2.5 μL of each primer, and PCR-grade water to adjust the volume. The PCR reaction procedure was as follows: predenaturation at 98°C for 30 s; denaturation at 98°C for 10 s, annealing at 54°C for 30 s, extension at 72°C for 45 s, with 32 cycles; and then final extension at 72°C for 10 min.

The PCR products were confirmed with 2% agarose gel electrophoresis, purified by AMPure XT beads (Beckman Coulter Genomics, Danvers, MA, USA), and quantified through Qubit (Invitrogen, USA). The amplicon pools were prepared for sequencing; furthermore, the size and quantity of the amplicon library were measured using Agilent 2100 Bioanalyzer (Agilent, USA) and with the Library Quantification Kit for Illumina (Kapa Biosciences, Woburn, MA, USA), respectively. The libraries were sequenced on the Illumina NovaSeq PE250 platform. Paired-end reads were assigned to the relevant samples on the basis of their unique barcode and truncated by cutting off the barcode and primer sequence and merged using FLASH (v1.2.11). To obtain the high-quality clean tags based on Fastp (v0.19.6), the raw reads were filtered for quality using specific filtering conditions. Chimeric sequences were removed using the Vsearch software (v2.3.4). After dereplication using DADA2, we obtained the feature table and sequence. Subsequently, feature abundance was normalized using the relative abundance of each sample according to SILVA (v138) classifier. Alpha and Beta diversity were calculated using Mothur (v1.30.2) and QIIME (v1.9.1). Additionally, the graphs were prepared using the R package. Blast was used for sequence alignment, and the feature sequences were annotated with the SILVA database for each representative sequence. Furthermore, a linear discriminant analysis (LDA) effect size (LEfSe) analysis was performed to compare species with significant intergroup differences (10). Functional profiles of microbial communities were predicted using PICRUSt2 (v2.2.0) (11).

### Metabolomic analysis

The sample preparation, metabolite identification and quantification, and primary quality control (QC) were performed at Majobio Bio-pharm Technology Co., Ltd (Shanghai, China) as described previously. After Ultra-high performance liquid chromatography–tandem mass spectrometry (HPLC-MS) analyses, the Progenesis QI 2.3 (Nonlinear Dynamics, Waters, USA) was used for peak detection and alignment of raw data. Subsequently, a data matrix was generated, which included retention time (RT), mass-to-charge ratio (m/z) values, and peak intensity. Metabolic features detected a minimum of 80% in any set of samples were retained. After filtering, quality control, normalization, and imputation, all data were log-transformed before statistical analysis. The metabolites were identified based on accurate mass, MS/MS fragments spectra, and isotope ratio difference in the human metabolome database (HMDB) (http://www.hmdb.ca/) and Metlin database (https://metlin.scripps.edu/).

The following composition analysis was implemented using the Majorbio cloud platform (https://cloud.majorbio.com). All multivariate statistical analyses, including principal component analysis (PCA) and partial least squares, discriminate analysis (PLS-DA), were performed using the “ropls” package for R software. Differentially expressed metabolites were identified using a combination of Variable importance in the projection (*VIP*) values of the PLS-DA model and Student's *t-test*. Metabolites with *VIP* > 2 and *P* < 0.05 indicated the presence of significant intergroup differences. The Kyoto Encyclopedia of Genes and Genomes (KEGG) pathway annotation and enrichment analysis were performed utilizing the KEGG pathway database (http://www.genome.jp/kegg). To test the co-linearity between metabolites, variance inflation factors (*VIF*) were used. The metabolites with *VIF* > 10 were removed before the redundancy analysis (RDA) analysis. The RDA and envfit analyses were performed using the vegan package in R software.

### Statistical analysis

Statistical analyses were performed with SPSS 22.0. The continuous data were presented as means + SEM or medians and interquartile ranges (IQRs). The student's *t*-test or one-way analysis of variance was used for the normally distributed data. *P* < 0.05 indicated statistical significance.

## Results

### Effects of FA on growth and slaughter performance

Compared with the control group, the ADG and FCR in the FA-H group increased and decreased by 21.32 and 14.46%, respectively; however, no significant differences (*P* > 0.05) were observed (Table 2). Furthermore, the four groups showed no intergroup differences (*P* > 0.05) in terms of ADFI, laying rate and feed/egg ratio.

**Table 2:** Effects of dietary supplementation of FA on growth performance of Xianju yellow chickens (Mean ± SD).

**Items**	**Control**	**FA-L**	**FA-M**	**FA-H**	***P-*value**
ADG (g)	2.101 ± 0.438	2.000 ± 0.931	2.290 ± 0.368	2.675 ± 0.947	0.411
ADFI (g)	93.400 ± 5.331	92.944 ± 9.160	89.849 ± 3.836	94.977 ± 3.799	0.487
FCR	46.012 ± 9.689	55.058 ± 25.118	39.966 ± 5.693	39.357 ± 13.893	0.300
Laying rate (%)	38.333 ± 6.731	37.421 ± 5.713	37.698 ± 3.514	37.619 ± 3.682	0.991
Feed/egg	4.990 ± 0.763	4.917 ± 0.763	4.897 ± 0.796	5.229 ± 0.516	0.819

As shown in Table 3, the general carcass characteristics such as dressed percentage, percentage of half-eviscerated yield with giblet, percentage of eviscerated yield, percentage of breast muscle yield, and percentage of leg muscle yield of chickens were similar regardless of supplement with varying concentration of FA (*P* > 0.05). However, the percentage of abdominal fat yields were different among FA supplement groups; FA-H vs. FA-L and FA-M (*P* < 0.05). In addition, the four groups showed no significant intergroup differences in terms of the length of the duodenum, jejunum, ileum, and ceca (*P* > 0.05) (Table 4). Breast muscle color and *pH* at 45 min after slaughter were no significant differences among the treatments (*P* > 0.05) (Table 5).

**Table 3:** Effects of dietary supplementation of FA on slaughter performance of Xianju yellow chickens (Mean ± SD).

**Items**	**Control**	**FA-L**	**FA-M**	**FA-H**	***P-*value**
Dressed percentage (%)	92.84 ± 1.38	91.31 ± 1.73	91.08 ± 2.24	91.82 ± 1.22	0.307
Percentage of half-eviscerated yield with giblet (%)	78.83 ± 2.04	77.68 ± 2.86	78.42 ± 2.73	75.81 ± 4.88	0.435
Percentage of eviscerated yield (%)	69.72 ± 2.08	66.41 ± 2.84	67.65 ± 2.09	67.37 ± 4.87	0.367
Percentage of breast muscle yield (%)	12.74 ± 1.93	13.67 ± 0.90	12.97 ± 1.37	12.93 ± 2.41	0.811
Percentage of leg muscle yield (%)	17.91 ± 1.82	18.40 ± 1.39	17.84 ± 0.69	17.8 ± 2.30	0.920
Percentage of abdominal fat yield (%)	7.20 ± 0.56^ab^	9.63 ± 1.47^a^	9.21 ± 1.47^a^	6.09 ± 2.30^b^	0.003

a, b *Within a column, means with different superscripts differ at P < 0.05*.

**Table 4:** Effects of dietary supplementation of FA on intestine length of Xianju yellow chickens (Mean ± SD).

**Items**	**Control**	**FA-L**	**FA-M**	**FA-H**	***P-*value**
Duodenum (cm)	11.05 ± 1.85	11.02 ± 1.51	10.68 ± 1.91	10.70 ± 1.45	0.970
Jejunum (cm)	49.73 ± 5.79	52.10 ± 8.15	50.00 ± 6.77	51.23 ± 4.13	0.912
Ileum (cm)	32.92 ± 4.08	30.33 ± 4.67	29.84 ± 1.46	34.12 ± 3.95	0.216
Ceca (cm)	16.37 ± 1.31	15.03 ± 2.34	15.30 ± 3.47	14.47 ± 2.10	0.575

**Table 5:** The pH and meat color at 45 min of breast muscle (Mean ± SD).

**Items**		**Control**	**FA-L**	**FA-M**	**FA-H**	***P-*value**
pH		6.72 ± 0.27	6.55 ± 0.34	6.75 ± 0.18	6.67 ± 0.30	0.631
Color	L*	58.39 ± 1.74	55.11 ± 3.20	56.3 ± 1.37	58.47 ± 5.97	0.308
	a*	8.87 ± 1.35	8.92 ± 3.23	10.21 ± 1.8	10.09 ± 1.63	0.563
	b*	5.87 ± 1.59	7.37 ± 2.47	6.97 ± 1.38	7.83 ± 1.74	0.323

L^*^, lightness of the meat color; a^*^, redness of meat; b^*^, yellow meat color.

### FA supplement in Xianju yellow chicken alters IL-2 and TNF-α, without influencing liver function indexes

**Figure 1:**
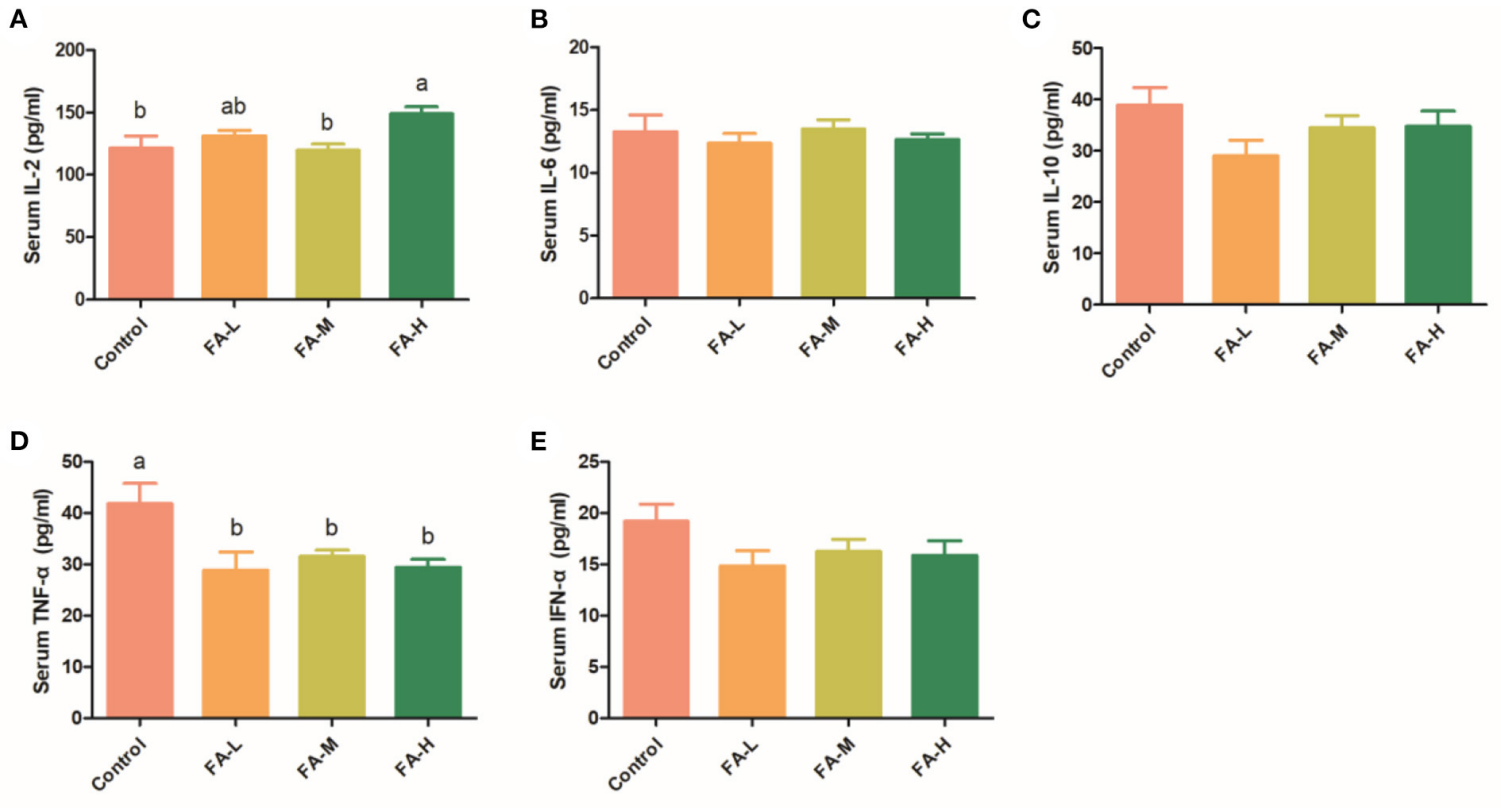
Effect of FA on serum concentration of IL-2 **(A)**, IL-6 **(B)**, IL-10 **(C)**, TNF-α **(D)**, and IFN-α **(E)**. Data with different superscript letters are significantly different (*P* < 0.05).

In serum, the level of IL-2 in the FA-H group was significantly higher than in the control and FA-M group (*P* < 0.05) (Figure 1A). Moreover, compared with the control group, supplementation of FA significantly decreased the serum levels of TNF-α (*P* < 0.05) (Figure 1D). Compared with the control, the levels of IL-6, IL-10, and IFN-α were relatively lower in chickens supplemented with FA (Figures 1B,C,E); however, the differences were not significant (*P* > 0.05).

**Figure 2:**
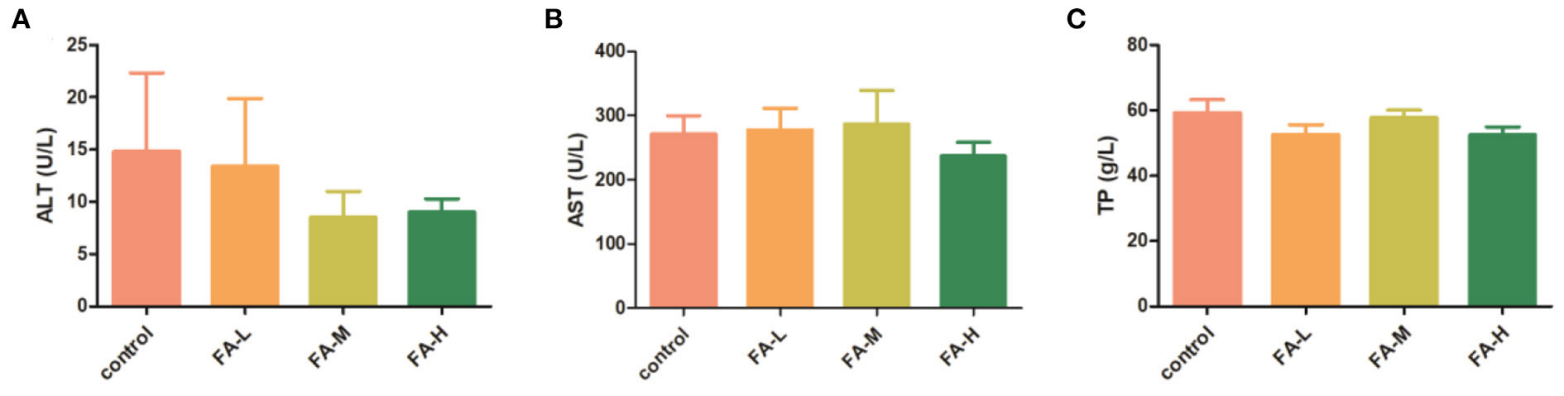
Effect of FA on serum ALT **(A)**, AST **(B)**, and TP **(C)**.

The liver function indexes were assayed to further evaluate the effects of FA in poultry. There was no significant difference in AST, ALT, and TP (Figure 2).

### FA supplement did not change *Alpha* diversity in the gut microbiota

For the control and FA-H samples, 1.198 million high-quality sequences (mean length, 419) were generated, representing 4,334 bacterial operational taxonomic units (OTUs) at 97% sequence similarity. As estimated by the *Alpha* diversity, no significant differences (*P* > 0.05) were observed in terms of the diversity, richness, and evenness between the control and FA-H groups (Supplementary Table 2).

### Difference in gut bacterial community compositions between control and FA-H groups

**Figure 3:**
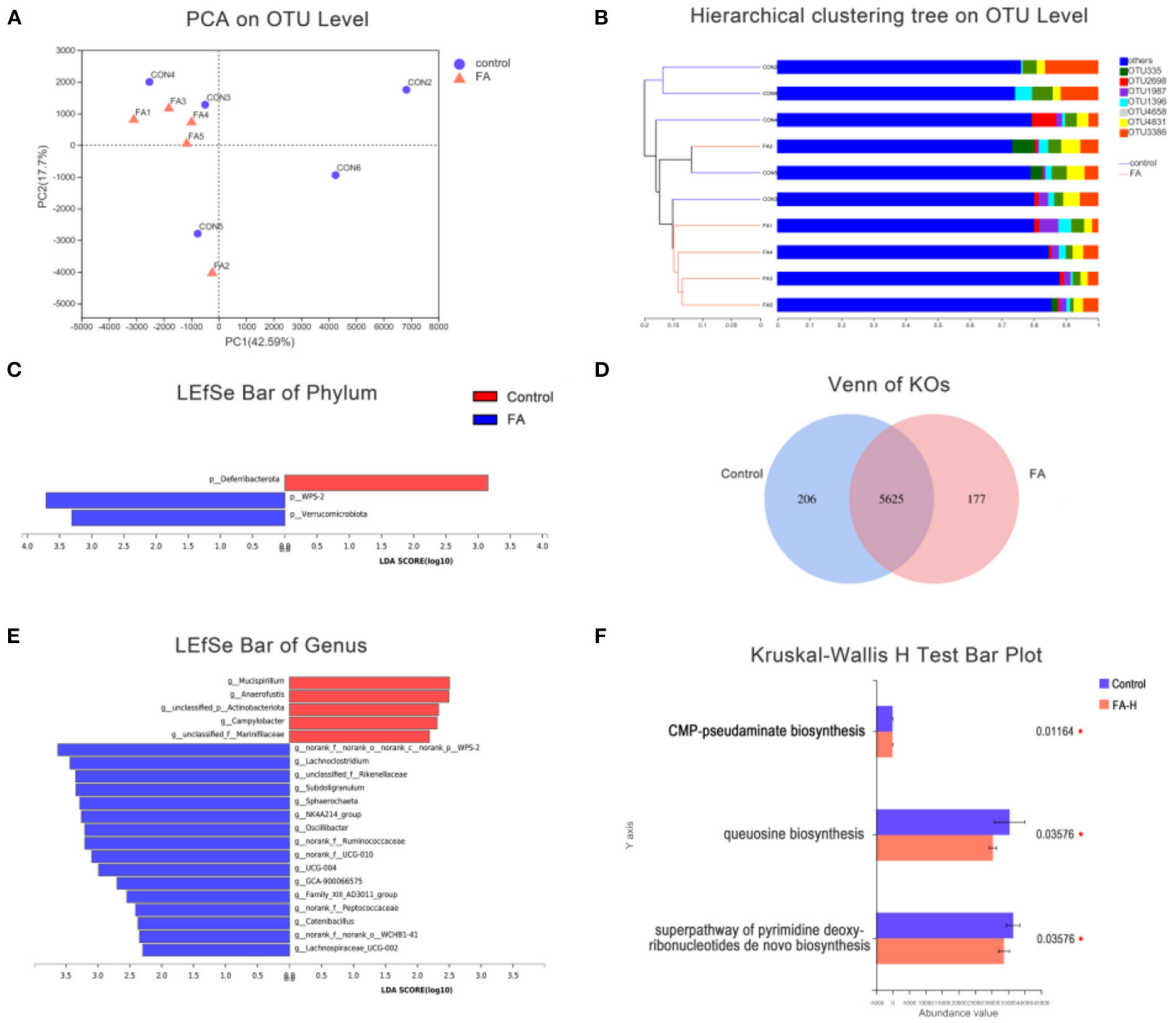
Effect of FA on gut microbiota. **(A)** PCA of gut microbiota communities based on OUT level. **(B)** Hierarchical sample clustering tree of the microbial community at OUT level based on Bray–Curtis distances. **(C)** Comparison of gut microbial variations at phylum level between control and FA-H group using LEfSe analysis. **(D)** Venn diagram indicated unique and shared KOs. **(E)** Comparison of gut microbial variations at genus level between control and FA-H group using LEfSe analysis. **(F)** Significantly enriched MetaCyc pathways of gut microbiota between the control and FA-H groups. *P < 0.05.

Regarding the β-diversity, PCA and Hierarchical Clustering Analysis revealed a powerful effect of FA. The first and second principal components of PCA clarified 42.59 and 17.7% of the total variation, respectively (Figure 3A). Additionally, some degree of clustering in FA-H samples was observed in the hierarchical clustering (Figure 3B). The LEfSe analysis was used to identify significant differences in microbiota between the control and FA-H groups from phylum to genus levels. At the phylum level, the two major bacterial groups identified in the FA-H group were Verrucomicrobiota and WPS-2, whereas Deferribacterota was highly represented in the control group (Figure 3C). There were 16 relatively high abundance genera in the FA-H group, including *Lachnoclostridium, Subdoligranulum, Sphaerochaeta, Oscillibacter*, and *Catenibacillus*, among others. Additionally, *Mucispirillum, Anaerofustis*, and *Campylobacter* were abundant in the control group (LDA > 2, *P* < 0.05) (Figure 3E).

To investigate the significant features of gut microbiota between the control and FA-H groups, PICRUSt2 was used to predict KEGG orthologous groups (KOs), MetaCyc pathways, and the abundance of Cluster of Orthologous Group of Proteins (COG). A total of 304 KOs were altered between the control and FA-H groups (Supplementary Table 3). Venn diagrams showed that 206 and 177 KOs were unique to the control and FA-H groups, respectively (Figure 3D). Three MetaCyc functional pathways, including “Superpathway of pyrimidine deoxyribonucleotides *de novo* biosynthesis,” “Queuosine biosynthesis,” and “CMP-pseudaminate biosynthesis,” were significantly enriched (*P* < 0.05) in the control group (Figure 3F). In the COG classification, 4,126 OTUs were classified into 25 function classifications. The “Function unknown” represented the richest function category, followed by “Amino acid transport and metabolism,” “Translation, ribosomal structure, and biogenesis,” and “Cell wall/membrane/envelope biogenesis” (Supplementary Figure 1).

### Alteration in metabolic signature between control and FA-H group

**Figure 4:**
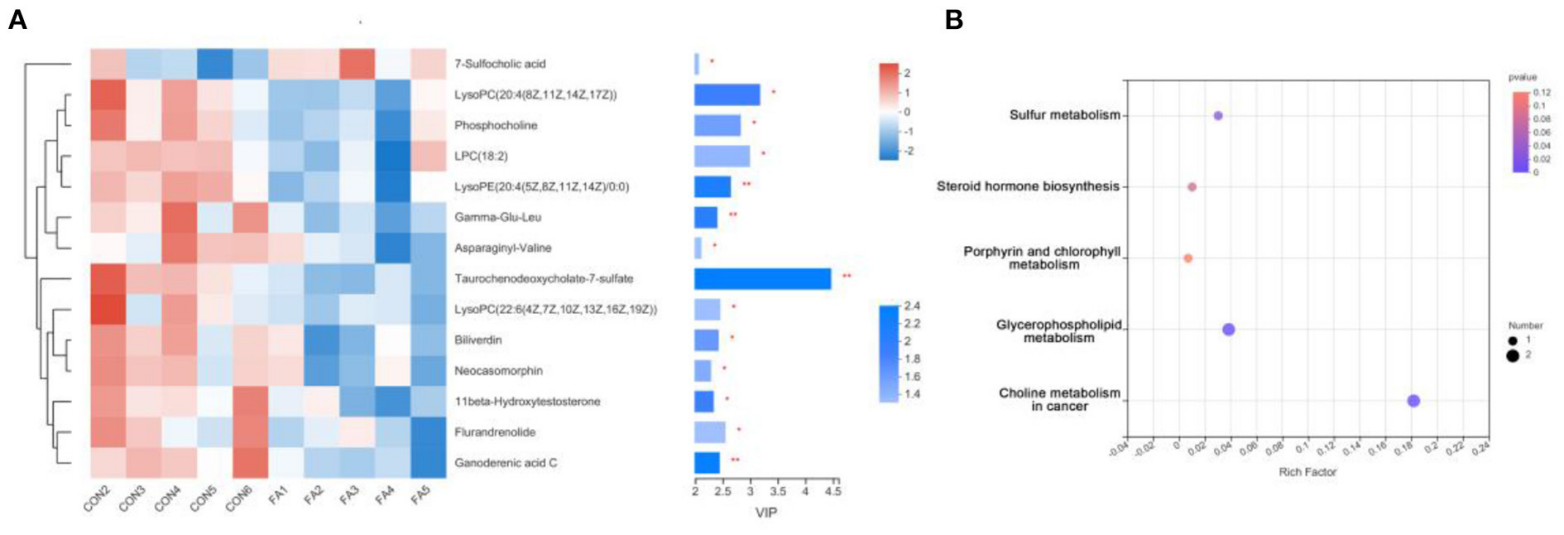
Effect of FA on gut metabolomic. **(A)** *VIP* scores with the expression of the heat map of intestinal metabolites in the FA-H and control groups. The selected metabolites were those with *VIP* ≥ 2 and *P* < 0.05. *P < 0.05, **P < 0.01. **(B)** Comparison of the KEGG enriched pathways of differential gut metabolites in the FA-H and in the control group. The size of the bubble represents the number of enriched metabolites, and the color indicates enrichment significance (*P-value*).

Using HPLC-MS, untargeted metabolite analysis was performed to investigate the alteration of the metabolome and examine the correlation between gut metabolites and microbiota. A total of 406 metabolites ions in both positive and negative ion modes were obtained from the extracted digesta samples of ceca. In addition, 14 annotated metabolites were significantly altered in the FA-H group compared to the control group (*P* < 0.05, PLS-DA VIP > 2) (Figure 4A; Supplementary Table 4). We performed receiver operating characteristic curve analysis on our data to evaluate the significance of metabolite discrimination. The area under the curve (AUC) was analyzed to assess the accuracy of potential biomarkers. Among the 14 differential metabolites, 12 showed an AUC of >0.85 with high sensitivity and specificity (Supplementary Figure 2). Therefore, 11β-Hydroxytestosterone, Asp-Ile, Biliverdin, Flurandrenolide, Gamma-Glu-Leu, Ganoderenic acid C, LysoPC 18:2, LysoPC 20:4 (8Z, 11Z, 14Z, 17Z), LysoPC 22:6 (4Z, 7Z, 10Z, 13Z, 16Z, 19Z), LysoPE 20:4 (5Z, 8Z, 11Z, 14Z)/0:0, Neocasomorphin, and Phosphocholine may be the key metabolites affected by FA.

The functions of these differential metabolites were determined using KEGG pathway analysis (Figure 4B). According to the analysis, 3 KEGG pathways, including “Sulfur metabolism,” “Glycerophospholipid metabolism,” and “Choline metabolism in cancer,” were identified (*P* < 0.05). Additionally, four significantly decreased metabolites including LysoPC 22:6 (4Z, 7Z, 10Z, 13Z, 16Z, 19Z), Phosphocholine, LysoPC 20:4 (8Z, 11Z, 14Z, 17Z), and 11β-Hydroxytestosterone were involved in lipid metabolism. These results indicated that FA supplementation may primarily affect lipid metabolism.

### Correlation analysis between intestinal microbiota and metabolites

**Figure 5:**
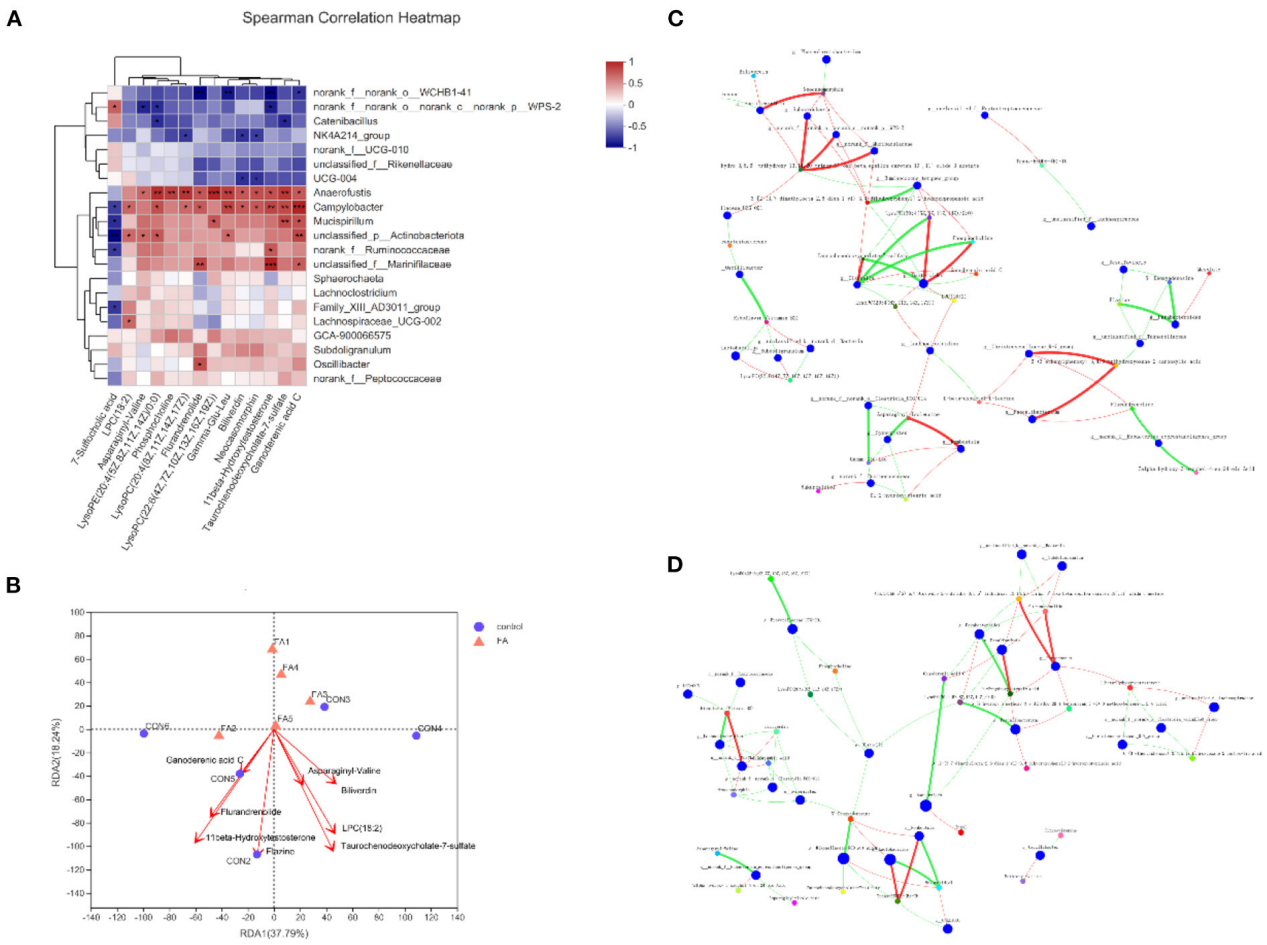
Correlation of gut metabolites and microbiota between FA-H and control group. **(A)** Heat map of correlation between differential metabolites and microbiota based on Spearman's correlation analysis. *P < 0.05, **P < 0.01, ***P < 0.001. **(B)** RDA plot showing the correlation between microbial community composition and metabolites. **(C)** Bivariate correlation analysis of microbiota and metabolites in FA-H group. **(D)** Bivariate correlation analysis of microbiota and metabolites in control. A connection represents a strong (Spearman's correlation coefficient [ρ] > 0.5) and significant (*P* < 0.01) correlation. Red indicates positive correlation, whereas green indicates negative correlation. The size of each node represents the relative abundance of the species.

To determine the association of changes in the gut microbiota and metabolites, we used the Spearman rank correlation analysis on the microbiota and metabolites that changed significantly after supplementation with FA (Figure 5A). Notably, nearly all differential metabolites showed a positive association with *Campylobacter* and *Anaerofustis*. After removing the redundant variables, seven metabolites were selected for RDA. The length of arrows in the RDA plot indicated the strength of the correlation between the environmental factor and sample distribution. RDA revealed that 11beta-Hydroxytestosterone, Flazine, and Taurochenodeoxycholate-7-sulfate exhibited the strongest correlation with microbiota composition (Figure 5B). However, no significant difference was found in the envfit analysis (Supplementary Table 5). To explore the key drivers involved in FA supplementation, we analyzed the co-occurrence analysis of microbiota and metabolites in the FA group. Base on the centralities of degree, closeness, and betweenness, the most important nodes of FA group were genus *Lachnoclostridium* and metabolite (3S, 5R, 6R, 6'S)-6,7-Didehydro-5,6-dihydro-3,5,6'-trihydroxy-13,14,20-trinor-3'-oxo-beta, epsilon-caroten-19',11'-olide 3-acetate (Figure 5C). Furthermore, the most important nodes of control were genus *Olsenella*, metabolites 5'-Deoxyadenosine and LysoPE 20:4 (5Z, 8Z, 11Z, 14Z)/0:0 (Figure 5D).

## Discussion

### Effects of FA on growth and slaughter performance in Xianju yellow chicken

Feed cost is consistently the largest expense on poultry production, which represents ~65–75%. Therefore, the FCR is an important index to measure economic benefits. Several independent studies have reported the improvement of livestock and poultry growth performance after the dietary supplementation of FA. Chand et al. (7) indicated that diets supplemented with FA improved growth performance of pigs by increasing ADG and feed ratio. Additionally, Mao et al. (12) observed that the addition of FA in broiler diets increased body weight gain. In the present study, we supplemented the chickens with 1,500 mg/kg FA in diet, although it did not significantly improve the growth performance of Xianju yellow chicken, we observed an increase in the ADG and a decrease in the FCR. And supply of 1,500 mg/kg FA cost about $4 per ton, which may not cause additional burden.

Dressed percentage and eviscerated percentage are important indicators for judging the slaughter performance. Our results showed that dressed percentage and eviscerated percentage of all groups reached 91 and 66%, respectively, which were similar to previous studies (13). Fat is widely known as a by-product that has a relatively low commercial value. Moreover, excessive deposition of abdominal fats is not only unfavorable to the health of poultry animals but also associated with reproductive dysfunction (14). We noted that the addition of FA significantly decreased the percentage of abdominal fat, which suggested that FA may affect the deposition of abdominal fat.

### Effects of FA on serum cytokine levels and liver function indexes in Xianju yellow chicken

The available literature contains little information on the immune-regulating effect of FA on the body. Our results have demonstrated that FA supplementation increased IL-2 levels and decreased TNF-α levels in serum, but did not affect IL-6, IL-10, and IFN-α levels. IL-2 is an important immune regulatory cytokine, and it can enhance T-cell proliferation, maintain T-cell function, and regulate the immune response function of T-cell, B-cell, NK cell, and monocytes (15). TNF-α is a well-known pro-inflammatory factor, and plays various roles in driving chronic inflammation and autoimmunity (16). TNF-α participates in vasodilation and edema formation, accelerates inflammation *via* increasing oxidative stress, and indirectly induces fever (17). Therefore, FA can regulate body immune response through activation of anti-inflammatory cytokines and suppression of pro-inflammatory cytokines.

The ALT and AST biochemical values were the main indicators for evaluating live health (18). As an enzyme, ALT is primarily present in cytosol, whereas AST is primarily present in mitochondria and cytoplasm in the liver. In cases of hepatocellular injury or alteration in the liver membrane permeability, ALT and AST are released into peripheral blood and elevate the serum levels of ALT and AST (19). Therefore, serum levels of ALT and AST are widely used as part of the routine diagnosis of liver disease. Serum TP may be elevated because of dehydration or diseases and reduced through impaired protein synthesis and increased protein loss (20). Owing to the fact that ALT, AST, and TP are reliable markers of liver function, our results revealed that the addition of FA at 1,500 mg/kg was safe for use and did not cause any disturbance to the liver functions of the chickens.

### Effects of FA on gut microbiota in Xianju yellow chicken

Gut microbiota are essential in maintaining gastrointestinal homeostasis and host health (21, 22). In the present study, we noted that *Lachnoclostridium* and *unclassified_f_Rikenellaceae* have increased relative abundance in the FA-H group, which showed an association with inflammation. *Lachnoclostridium* is a genus of gram-positive, obligate anaerobic, spore-forming, motile bacteria, which ferment mono- and disaccharides to produce acetate (23). The relative abundance of *Lachnoclostridium* was reportedly reduced in DSS-induced mice (24, 25), and negatively related to IL-6 (26). Rikenellaceae, comprises two genera, *Alistipes* and *Rikenella* (27). The proportion of Rikenellaceae was positively associated with TLR3, TLR8, sIgA, and IgG (28, 29). Our results demonstrated that FA could influence the specific microbes, which in turn affected immune regulation and intestinal homeostasis.

Furthermore, an increased abundance of several SCFA-producing genera, including *Subdoligranulum, Sphaerochaeta*, and *Catenibacillus*, were observed in the FA-H group. *Subdoligranulum*, which was isolated from human feces, is strictly anaerobic, gram-negative, and negative in terms of the intestinal permeability of the host (30, 31). Different from other members of phylum Spirochaetes, *Sphaerochaeta* genomes lacked the genes about motility and associated signal transduction; however, they are highly enriched in genes related to fermentation and carbohydrate metabolism (32). Consequently, they grow as biofilms and by fermenting carbohydrates to formate, acetate, propionate, and iso-butyrate (33, 34). *Catenibacillus* is a genus of anaerobic gram-positive bacteria and typically appears in the form of chains (35). Previous studies have shown that *Catenibacillus* could deglycosylate by not only deglycosylating puerarin to daidzein but also other aromatic C-glucosides and flavonoid O-glucosides (36). Their catabolites, protocatechuic acid, 3,4-dihydroxyphenylacetic acid, and 3,4-dihydroxyphenylpropionic acid, have been verified to exert anti-inflammatory and antioxidant effects by decreasing the level of pro-inflammatory cytokines IL-1β, IL-6, and TNF-α and reducing the contents of NO, H_2_O_2_, and malondialdehyde (37–39). These results implied that FA facilitated the abundance of SCFA-producing bacteria, and in turn regulated body fat metabolism and energy production.

*Mucispirillum, Anaerofustis*, and *Campylobacter* were three bacteria that were found enriched in the control birds. *Mucispirillum* colonizes in the intestinal mucus layer and exhibits the ability to degrade mucin (40). Although *Mucispirillum schaedleri* can assist the host's defenses against gut inflammation induced by Salmonella Typhimurium infection (41), most studies have demonstrated that several disease conditions were associated with *Mucispirillum*, including Crohn's disease, pathogen infection, and Parkinson's disease (42). Multiple studies have demonstrated that *Mucispirillum* was enriched during active colitis, and it has been identified as an indicator for colitis in the model of colitis (43, 44). *Anaerofustis* is a strictly anaerobic and gram-positive genus belonging to the family of Eubacteriaceae (45, 46) reported that *Anaerofustis* was enriched in heat-stressed broilers. In addition, stress susceptibility was associated with the increase of *Anaerofustis* in rats (47). *Campylobacter* is one of the most common bacterial causes of diarrhea worldwide; poultry is known to be the major source of this organism. Previous research supported that *Campylobacter* usually develops as an asymptomatic infection in poultry (48). However, recent studies have challenged this point and argue that *Campylobacter* disturbs poultry performance by impairing intestinal integrity, increasing intestinal permeability and bacterial translocation, and enhancing inflammatory response (49). Our results indicated that FA might promote poultry health by reducing the abundance of pathogenic microorganisms.

### Effects of FA on gut metabolome in Xianju yellow chicken

Phosphocholines are storage forms for choline within the cytosol. Studies have reported that phosphocholines were associated with cell and hepatic regeneration, and significantly elevated in acute hepatitis and liver injury (50, 51). Chenodeoxycholic acid, cholic acid, and b biliverdin are the main components of bile in poultry. The decreased amounts of taurochenodeoxycholate-7-sulfate and biliverdin in the intestinal tract may indicate that FA is involved in bile acid circulation. However, the underlying specific mechanisms are required for further investigation. Notably, the amount of some dipeptides and peptides, including γ-Glu-Leu, Asp-Val, and neocasomorphin, were significantly decreased in the FA group, which might be related to reduced microbial abundance.

## Conclusion

The present study suggests the use of novel feed additives such as FA in poultry production. On the basis of our finding, FA at the level of 1,500 mg/kg into the diet of Xianju yellow chicken could enhance growth performance, improve immune function, and modulate intestinal microbiota community and metabolites.

## Data availability statement

The data presented in the study are deposited in the NCBI SRA BioProject repository, and the accession number is PRJNA841423.

## Ethics statement

The animal study was reviewed and approved by the Ethics Committee of the Zhejiang Academy of Agricultural Sciences.

## Author contributions

PF: conceptualization, methodology, formal analysis, investigation, and writing-original draft. QL: software and formal analysis. HS and JG: investigation. XY: resources. YT: writing-review and editing and funding acquisition. PW: project administration and funding acquisition. All authors contributed to the article and approved the submitted version.

## Funding

This study was financially supported by the Key Research and Development Program of Zhejiang Province (Nos. 2022C03050 and 2021C02034) and Zhejiang Provincial Special Commissioner Team Projects of Science & Technology (No. Xianju Chicken Industry, 2020-2024).

## Conflict of interest

Author XY was employed by the company Xianju Breeding Chicken Farm, Taizhou, China. The remaining authors declare that the research was conducted in the absence of any commercial or financial relationships that could be construed as a potential conflict of interest.

## Publisher's note

All claims expressed in this article are solely those of the authors and do not necessarily represent those of their affiliated organizations, or those of the publisher, the editors and the reviewers. Any product that may be evaluated in this article, or claim that may be made by its manufacturer, is not guaranteed or endorsed by the publisher.

## Supplementary Material

Supplementary Figure 1 COG function classification of the control and FA-H groups.

Supplementary Figure 2 The ROC analysis of differential metabolites between control and FA-H group.
