# Supplementary figures and images for: Effects of fulvic acid on growth performance, serum index, gut microbiota, and metabolites of Xianju yellow chicken
Source: Front Nutr. 2022 Aug 5;9:963271. doi: 10.3389/fnut.2022.963271 (PMC9389313; doi:10.3389/fnut.2022.963271)

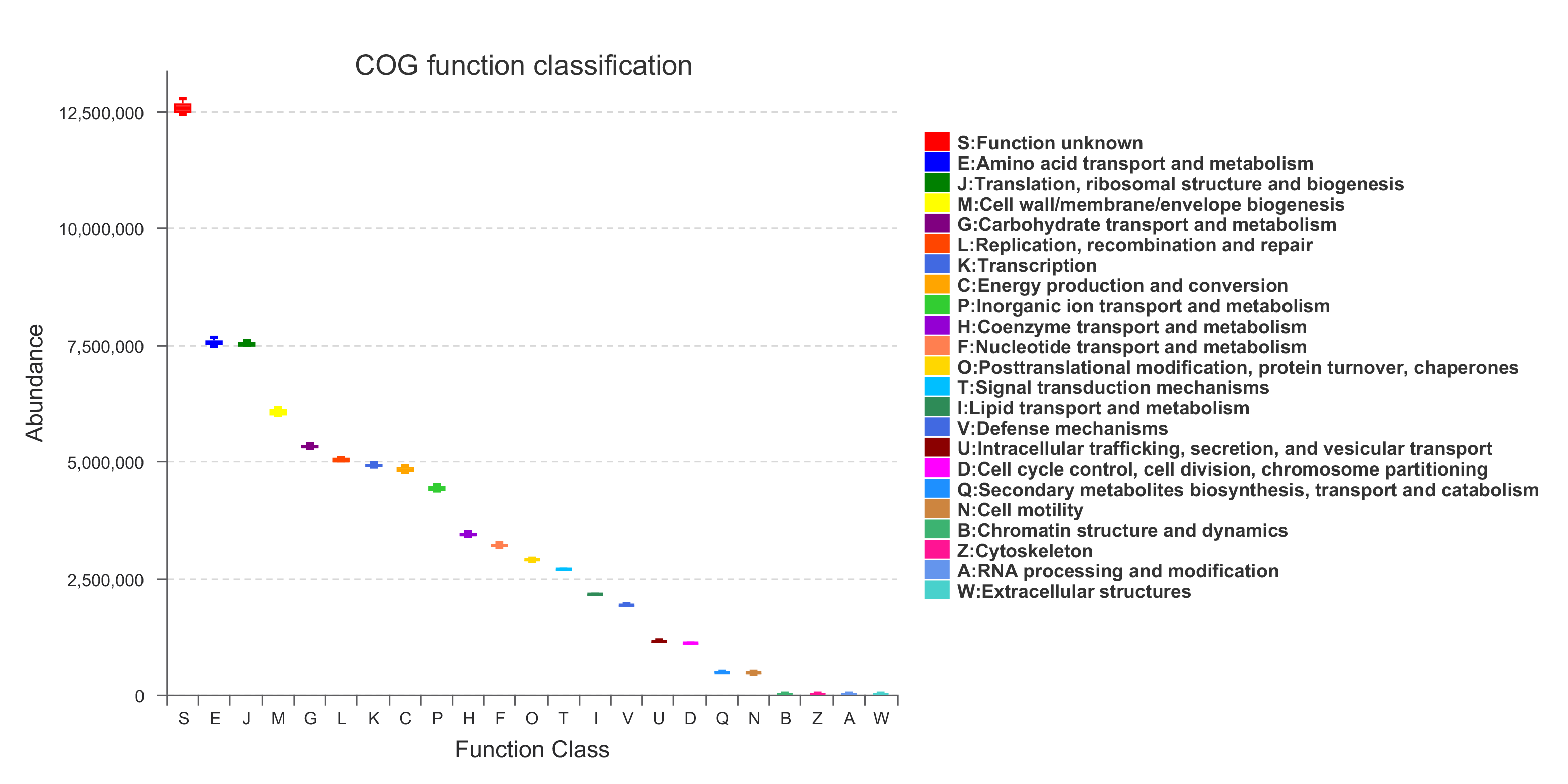

Supplement: Supplementary Figure 1 — COG function classification of the control and FA-H groups. [file Image_1.TIF]

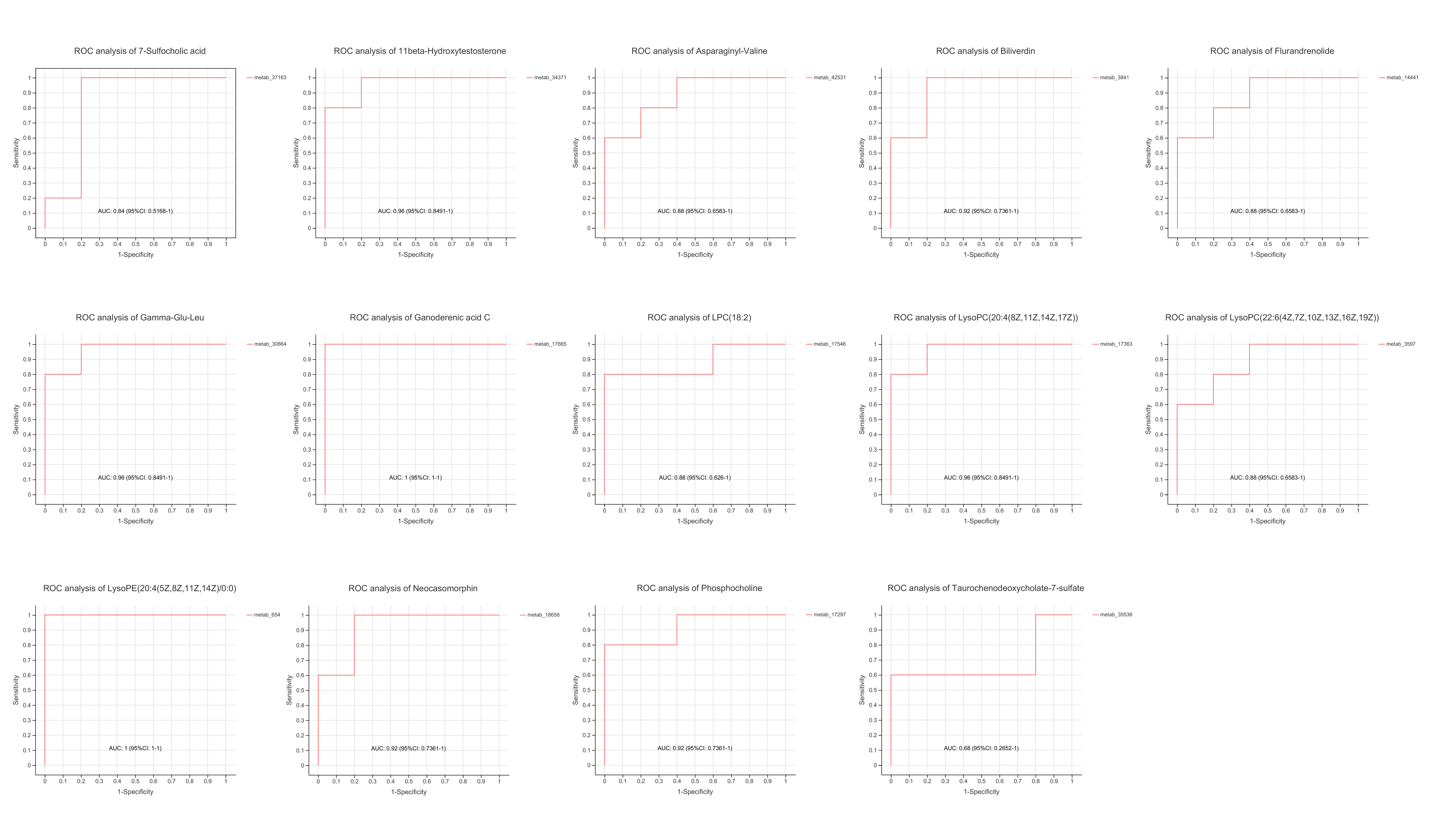

Supplement: Supplementary Figure 2 — The ROC analysis of differential metabolites between control and FA-H group. [file Image_2.TIF]
