# Supplementary material for: Effects of fulvic acid on growth performance, serum index, gut microbiota, and metabolites of Xianju yellow chicken
Source: Front Nutr. 2022 Aug 5;9:963271. doi: 10.3389/fnut.2022.963271 (PMC9389313; doi:10.3389/fnut.2022.963271)
Supplement: Supplementary file 3 [file Table_1.DOCX]

Supplementary table 1 The composition and hygienic index of FA

| Item | Content |
| --- | --- |
| Fulvic acid | 19.28% |
| Moisture | 11.78% |
| Total arsenic | ≤ 10 mg/kg |
| Lead, Pb | ≤ 15 mg/kg |
| Mercury, Hg | ≤ 0.1 mg/kg |
| Cadmium, Cd | ≤ 2 mg/kg |
| Chromium, Cr | ≤ 5 mg/kg |
| Fluorine, F | ≤ 400 mg/kg |
| Nitrite | ≤ 15 mg/kg |
| Polychlorinated biphenyls, PCBs | ≤ 10 mg/kg |
| Hexachlorocyclohexane, BHC | Not detected |
| Dichlorodiphenyltrichloroethane, DDT | Not detected |
| Hexachlorobenzene, HCB | Not detected |
