# Supplementary material for: Effects of fulvic acid on growth performance, serum index, gut microbiota, and metabolites of Xianju yellow chicken
Source: Front Nutr. 2022 Aug 5;9:963271. doi: 10.3389/fnut.2022.963271 (PMC9389313; doi:10.3389/fnut.2022.963271)
Supplement: Supplementary file 4 [file Table_2.DOCX]

| Estimators | Control | FA | Pvalue |
| --- | --- | --- | --- |
| sobs | 1417.80±102.06 | 1476.60±54.70 | 0.6761 |
| shannon | 5.068±0.242 | 5.362±0.166 | 0.0601 |
| simpson | 0.022±0.011 | 0.013±0.004 | 0.0601 |
| ace | 1672.50±113.48 | 1714.60±51.70 | 0.8345 |
| chao | 1573.40±113.01 | 1622.60±52.57 | 0.8345 |
| shannoneven | 0.698±0.0268 | 0.735±0.0202 | 0.0601 |
| simpsoneven | 0.036±0.0114 | 0.056±0.0123 | 0.0601 |

Supplementary Table 2 Alpha diversity in gut microbiota of the control and FA group
